# Divergence in drought‐response traits between sympatric species of *Mimulus*

**DOI:** 10.1002/ece3.5549

**Published:** 2019-08-14

**Authors:** Samuel J. Mantel, Andrea L. Sweigart

**Affiliations:** ^1^ Department of Genetics University of Georgia Athens GA USA

**Keywords:** drought, ecological isolation, flowering time, speciation, sympatry

## Abstract

Differential adaptation to local environmental conditions is thought to be an important driver of speciation. Plants, whose sedentary lifestyle necessitates fine‐tuned adaptation to edaphic conditions such as water availability, are often distributed based on these conditions. Populations occupying water‐limited habitats may employ a variety of strategies, involving numerous phenotypes, to prevent and withstand desiccation. In sympatry, two closely related *Mimulus* species—*M. guttatus* and *M. nasutus*—occupy distinct microhabitats that differ in seasonal water availability. In a common garden experiment, we characterized natural variation within and between sympatric *M. guttatus* and *M. nasutus* in the ability to successfully set seed under well‐watered and drought conditions. We also measured key phenotypes for drought adaptation, including developmental timing, plant size, flower size, and stomatal density. Consistent with their microhabitat associations in nature, *M. nasutus* set seed much more successfully than *M. guttatus* under water‐limited conditions. This divergence in reproductive output under drought was due to differences in mortality after the onset of flowering, with *M. nasutus* surviving at a much higher rate than *M. guttatus*. Higher seed set in *M. nasutus* was mediated, at least in part, by a plastic increase in the rate of late‐stage development (i.e., fruit maturation), consistent with the ability of this species to inhabit more ephemeral habitats in the field. Our results suggest adaptation to water availability may be an important factor in species maintenance of these *Mimulus* taxa in sympatry.

## Graphical abstract

Differential adaptation to local environmental conditions such as water availability has been implicated as an important driver of speciation. Consistent with the microhabitats sympatric *Mimulus* species are known to inhabit, seed set in these species diverges dramatically under drought conditions. Thus, adaptation to water availability may be an important factor in the maintenance of these species in the face of interspecific gene flow.

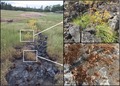

## 1 INTRODUCTION

Reciprocal transplant experiments in diverse taxa have often shown that organisms are strongly adapted to their local environments (Hereford, 2009; Leimu & Fischer, 2008). Over time, divergent selection among these distinct environments can lead to reproductive isolation (Rundle & Nosil, 2005; Schluter, 2000), either by preventing closely related species from coming together at all (i.e., geographic isolation) or by limiting opportunities for interspecific gene flow if they do. In the latter case, when closely related species occur sympatrically, adaptation to different microhabitats can promote premating isolation if species are spatially or temporally separated, and/or extrinsic postzygotic isolation if hybrid progeny suffer a fitness disadvantage in parental habitats (Coyne & Orr, 2004). Although adaptation to different habitats is thought to be one of the most important drivers of speciation (Sobel, Chen, Watt, & Schemske, 2010), in most cases, little is known about the ecological factors involved or the particular phenotypes that contribute to divergence.

In plants, water availability is a key determinant of species distributions (Cornwell & Grubb, 2003; Engelbrecht et al., 2007) and heterogeneity in soil moisture is often associated with local adaptation within species (Clausen, Keck, & Hiesey, 1940; Hall & Willis, 2006; Kooyers, Greenlee, Colicchio, Oh, & Blackman, 2015; Lasky et al., 2014, 2012; Lee & Mitchell‐Olds, 2013). To succeed in water‐limited environments, plants have evolved a diverse array of physiological, developmental, and life history adaptations (Bartels & Sunkar, 2005; Kooyers, 2015; Maggio, Zhu, Hasegawa, & Bressan, 2006). These traits are often categorized into three strategies—those that enable plants to escape, avoid, or tolerate drought conditions (Kooyers, 2015; Ludlow, 1989). In an escape strategy, plants typically develop rapidly and reproduce prior to drought‐induced senescence. In contrast, with avoidance and tolerance strategies, plants prevent drought‐induced senescence by increasing water‐use efficiency (e.g., via a decrease in stomatal conductance) or though physiological changes (e.g., osmotic adjustment, root growth). Because these strategies involve diverse mechanisms and suites of traits, adaptation to dry soils is often accompanied by dramatic phenotypic changes, which can have important consequences for reproductive isolation between closely related sympatric species. For example, a shift to earlier flowering—a hallmark of drought escape—can lead to phenological isolation (Fishman, Sweigart, Kenney, & Campbell, 2014; Franks & Weis, 2009; Martin, Bouck, & Arnold, 2005). Despite the potential importance of water availability as an axis of plant divergence, there are few detailed characterizations of drought adaptation between closely related species that grow sympatrically (Dunning et al., 2016; Eckhart, Geber, & McGuire, 2004; Geber & Eckhart, 2005).

Here, we focus on divergence in drought response traits between the yellow monkeyflowers *Mimulus guttatus* and *M. nasutus*. *Mimulus guttatus* is a phenotypically and genetically diverse, primarily outcrossing species that occupies wet soils across western North America (Wu et al., 2008). *Mimulus nasutus* is a highly selfing species that diverged recently (~200KYA) from *M. guttatus* (Brandvain, Kenney, Flagel, Coop, & Sweigart, 2014). The two species are largely allopatric, but sympatric populations of *M. nasutus* and annual ecotypes of *M. guttatus* are not uncommon throughout their shared range. In addition to their divergent mating systems and associated floral traits (Fishman, Kelly, & Willis, 2002), the two species show clear ecological differentiation, with *M. nasutus* flowering earlier and tending to occupy microhabitats that dry out sooner than *M. guttatus* (Kiang & Hamrick, 1978). This shift to earlier flowering in *M. nasutus* is caused, at least in part, by an ability to flower under much shorter day lengths (<10 hr) than *M. guttatus*, which often requires at least 14 hr of daylight to initiate reproduction (Friedman & Willis, 2013; Kooyers et al., 2015). When the two species co‐occur, divergence in critical photoperiod (Fishman et al., 2014), and in flowering phenology more generally, is a major barrier to interspecific mating (Kenney & Sweigart, 2016; Kiang & Hamrick, 1978; Martin & Willis, 2007). Despite this strong barrier, hybridization between sympatric populations of *M. guttatus* and *M. nasutus* can be substantial (Kenney & Sweigart, 2016) and there is clear evidence of ongoing interspecific introgression (Brandvain et al., 2014; Kenney & Sweigart, 2016; Sweigart & Willis, 2003).

**Figure 1:**
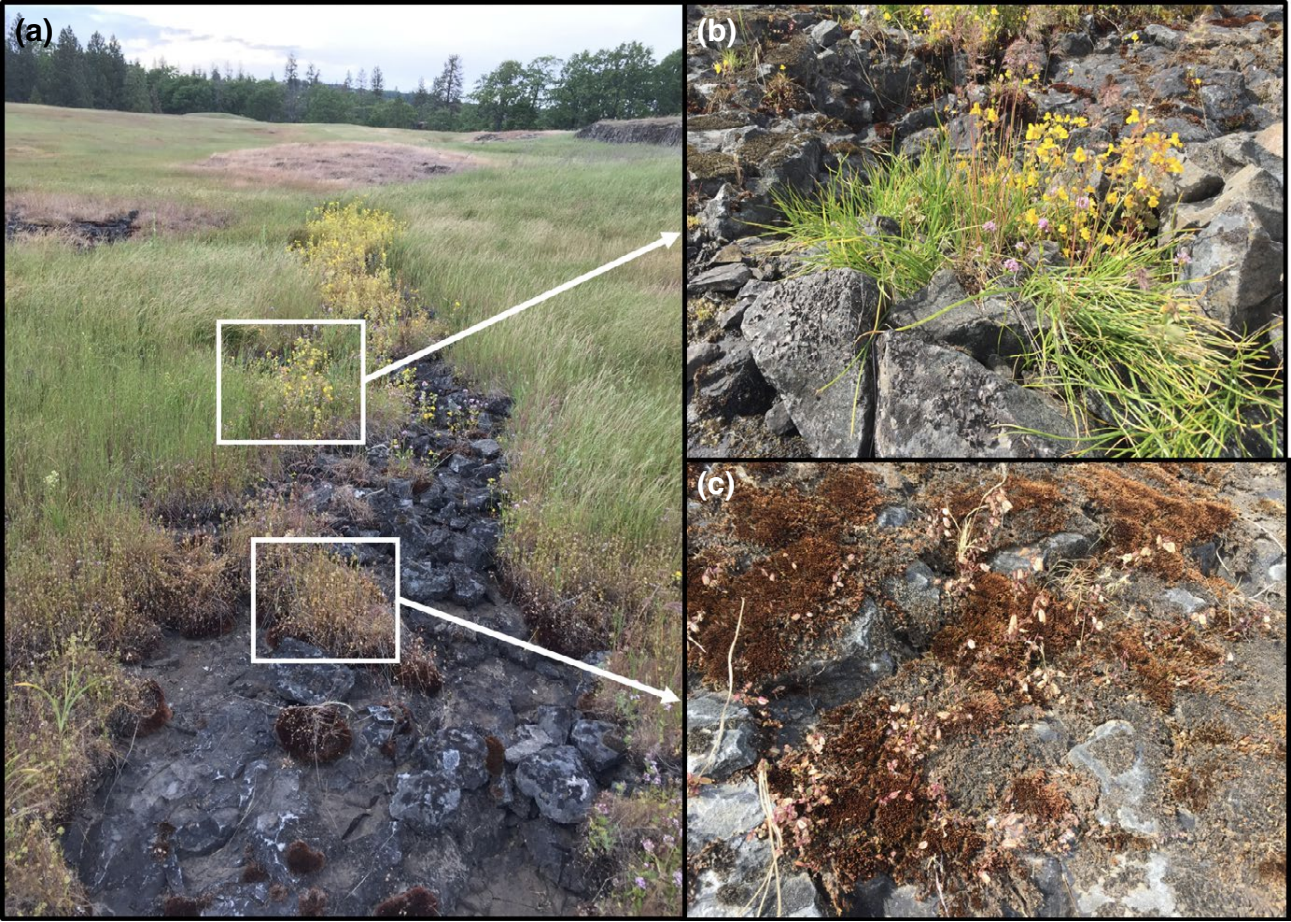
One of the streambeds of Catherine Creek (a) in May, with hybrid individuals and putative *Mimulus guttatus* (b) and *M. nasutus* (c) growing in close proximity, often within one meter

How, then, are these two *Mimulus* species maintained in the face of considerable gene flow? In a previous study (Kenney & Sweigart, 2016), we began to address this question by focusing on populations of *M. guttatus* and *M. nasutus* that have come into secondary contact at Catherine Creek (CAC), a gradually sloping, rocky meadow with streams and seeps that flow down into the Columbia River Gorge (Figure 1). Edaphic conditions, including water availability, are highly heterogeneous at this site, and although the two *Mimulus* species often grow within a meter of each other, they are found in somewhat distinct microhabitats: *M. nasutus* occurs in patches of moss in and around flowing streams that dry up in late spring, whereas *M. guttatus* grows in deeper seeps that stay wet through spring and into summer. The two species also flower asynchronously at the CAC site (Kenney & Sweigart, 2016) due, in part, to divergence at two major genetic loci for critical photoperiod (Fishman et al., 2014). The ability to flower under short days is likely a key drought escape strategy for CAC *M. nasutus*, allowing most individuals to complete reproduction by late May before ephemeral sources of water (snow melt and rain) are depleted. Nevertheless, even with species divergence in critical photoperiod, there is substantial overlap in the flowering phenologies of CAC *M. nasutus* and *M. guttatus*, as well as a large number of genetically admixed individuals that flower at intermediate times (Kenney & Sweigart, 2016). There is also ongoing introgression at CAC, mostly from *M. nasutus* into *M. guttatus* (Brandvain et al., 2014), including at one of the two mapped critical photoperiod loci (Kenney & Sweigart, 2016).

Given these observations at CAC, a key question is whether introgression from *M. nasutus* might facilitate drought escape in *M. guttatus*. The answer depends to some degree on whether drought adaptation is mediated by a relatively simple shift to earlier flowering (via a decrease in critical photoperiod) or requires a more complex, coordinated set of traits. Do the latest‐flowering *M. nasutus* and hybrids have additional mechanisms to deal with the onset of terminal drought? If an *M. guttatus* individual happens to germinate in a dry microsite, does it have any adaptations that might allow it to survive and reproduce? If an *M. guttatus* seedling carries an introgressed, photoperiod response allele from *M. nasutus*, would it have high fitness in a dry site, or are additional traits needed? Previous work has shown that closely related *Mimulus* taxa, including populations of *M. guttatus* and *M. nasutus*, are differentiated by a variety of drought escape and avoidance traits (Ivey & Carr, 2012; Kiang & Hamrick, 1978; Kooyers et al., 2015; Wu, Lowry, Nutter, & Willis, 2010), but little is known about variation in these phenotypes within and between sympatric populations. This issue is of fundamental importance for understanding species maintenance: if multiple traits (and genetic loci) are needed for drought adaptation, microhabitat isolation might be a potent barrier between species, even with considerable gene flow.

In this study, we performed a common garden experiment to investigate the phenotypic basis of microhabitat isolation between sympatric *M. guttatus* and *M. nasutus*. Using inbred lines derived from the sympatric CAC site and other natural populations, we grew plants under water‐limited conditions to simulate the onset of summer drought experienced by both *Mimulus* species across their native ranges. Because we were interested in exploring traits related to drought response beyond critical photoperiod, we grew all plants under inductive light conditions (16‐hr days). First, we examined the overall impact of drought on growth and fitness within and between species. Next, we dissected the phenotypic basis of dramatic differences in seed set between *M. guttatus* and *M. nasutus* under drought, exploring vegetative and reproductive traits, developmental rates, and survival across the life cycle. Is the larger flowered *M. guttatus* more vulnerable to desiccation (e.g., Dudley, Arroyo, & Fernández‐Murillo, 2018; Galen, Sherry, & Carroll, 1999) due to increased floral input? Do *Mimulus* taxa show variation in leaf traits related to water use efficiency? Do developmental rates vary, allowing for different levels of drought escape/avoidance? Strikingly, we discovered that *M. nasutus* alone is capable of accelerating its developmental rate in response to drought. This developmental shift, which occurs late in the life cycle (i.e., after flowering), has likely played a key role in adaptation within *M. nasutus* and contributed to divergence between species.

## 2 MATERIALS AND METHODS

### 2.1 Plant lines and growth conditions

To characterize natural variation in drought response within and between sympatric *M. guttatus* and *M. nasutus,* we generated a collection of inbred lines derived from CAC (Table 1). We produced 10 *M. guttatus* lines from wild‐collected CAC seed through at least four generations of self‐fertilization with single seed descent. To minimize maternal effects, we also propagated four CAC *M. nasutus* lines, from already naturally inbred wild‐collected seed, in the greenhouse for at least two generations. In addition to these lines from CAC, we included another five well‐characterized inbred lines (SWB38A, DUN10, IM767, DPRG102, and SF5) that have been involved in previous studies of ecological adaptation including drought response (Friedman, Twyford, Willis, & Blackman, 2015; Mojica, Lee, Willis, & Kelly, 2012; Wu et al., 2010; Table 1). These previously studied lines include coastal perennial and annual inland ecotypes of *M. guttatus*, which have been recognized as distinct taxonomic groups (Lowry, Rockwood, & Willis, 2008; Pennell, 1947), as well as one population of *M. nasutus*. In total, we grew 19 inbred lines: five *M. nasutus* lines (four from CAC), 12 annual *M. guttatus* lines (10 from CAC), and two perennial *M. guttatus* lines.

**Table 1:** Geographic locations of *Mimulus* populations used

Species/ecotype	Line abbreviation	Population	Latitude (N)	Longitude (W)
perennial *M. guttatus*	SWB38A	Sperm Whale Beach, Mendocino County, California	39°02′09″	123°41′25″
	DUN10	Oregon Dunes National Recreation Area, Lane County, Oregon	43°53′35″	124°08′16″
annual *M. guttatus*	IM767	Iron Mountain, Highway 20, Linn County, Oregon	44°24′03″	122°08′16″
	DPR102	Stanislaus National Forest Junction of Highway 120 and Jacksonville Road, Tuolumne County, California	37°49′45″	120°20′41″
	CAC6	Catherine Creek, Washington side of the Columbia River Gorge off of Hwy. 14	45°42′42″	121°21′55″
	CAC110			
	CAC112			
	CAC134			
	CAC141			
	CAC162			
	CAC171			
	CAC262			
	CAC277			
	CAC415			
*M. nasutus*	SF5	Sherar's Falls, Tygh Valley, Wasco County, Oregon	45°15′52″	121°01′21″
	CAC9	Catherine Creek, Washington side of the Columbia River Gorge off of Hwy. 14	45°42′43″	121°21′55″
	CAC22			
	CAC27			
	CAC32			

Seeds were planted into 2.5” pots filled with moist Fafard 3‐B potting mix (Sun Gro Horticulture), chilled for seven days at 4°C to promote germination, and moved to a Conviron growth chamber with lights set to 16‐hr days and temperatures set to 23°C days/16°C nights. For all temporal measurements, we set this day, when pots were moved to the growth chamber, as Day 0. Roughly a week after being moved to the growth chamber, seeds began to germinate, but exact germination dates were not recorded. Two to three days following germination (Days 8 and 9), we transplanted seedlings into 54 × 28 cm flats (Kord, HC Companies Canada), with holes for drainage, filled with moist Fafard 3‐B potting mix and moved them into the UGA greenhouses under 16‐hr supplemental light, 23°C days/16°C nights. Midway through the experiment, we discovered that the plants were likely experiencing low levels of 24‐hr light from an adjacent greenhouse, which might have contributed to overall faster flowering times (once discovered, we set these adjacent lights to 16‐hr days coordinated with our experiment). However, because plants were randomized within their blocks and all plants experienced the same conditions, no systematic bias was introduced. After transplanting, flats were bottom‐watered to saturation for five days to allow seedlings to recover from transplant and acclimate within their experimental blocks.

### 2.2 Experimental design

We grew plants under two distinct watering treatments to examine plant responses to variation in soil moisture conditions. Following transplant on Day 8 or 9 and the five‐day establishment period (Days 10–14), we initiated two treatments on Day 15: (a) well‐watered, in which we bottom‐watered flats daily to maintain soil saturation, and (b) dry‐down, in which we simulated the onset of seasonal drought by withholding all additional water and allowing flats to progressively dry from saturation.

To ensure that all plants within each treatment‐experienced similar levels of soil moisture, we grew plants together in large experimental flats, rather than in individual pots. With this design (modified from Wu et al., 2010), our intention was to minimize variation in soil drying rates due to plant size differences (i.e., larger plants may use water more quickly, reducing soil moisture). In each of the 76 blocks (38 per treatment), nine focal experimental plants were evenly spaced into a 2 × 5 grid and surrounded by "edge plants" from the IM767 inbred line of *M. guttatus* (9 experimental plants × 38 flats = 342 total plants per treatment; note that one position in each 2 × 5 grid was left vacant to monitor soil drying rate). With this design, each focal experimental plant was surrounded by eight or (when situated adjacent to the vacant position) seven neighboring plants. Within the two treatments, we randomized the positions of replicates from each of the 19 inbred lines (comprising three groups: *M. nasutus*, annual *M. guttatus*, and perennial *M. guttatus*) across the 38 blocks (average number of replicates per inbred line in each treatment = 18, range = 9–32). We began experimental treatments on Day 15 when we detected buds on 32 plants (15 in the well‐watered and 17 in the dry‐down treatment; 72% of these were from the *M. nasutus* line CAC32).

**Figure 2:**
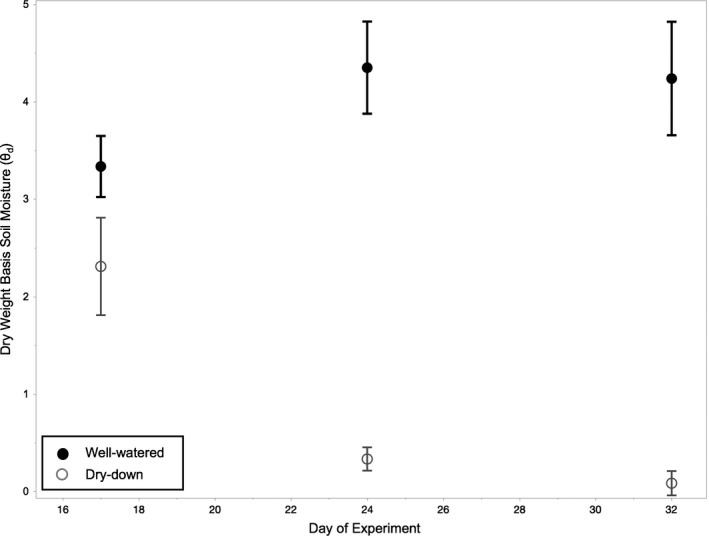
Dry weight basis soil moisture (θ_d_) in each watering treatment over the course of the experiment. Average soil moisture (error bars, *SE*) in well‐watered flats remained constant or increased over the course of the experiment, while dry‐down flats experienced continually decreasing soil moisture as the experiment progressed

For each experimental flat, we measured dry basis soil moisture content (θ_d_) on Days 17, 24, and 32 (Day 0 was when seeds were removed from stratification, watering treatments were initiated on Day 15). To perform this measurement, we took soil samples from the vacant positions and recorded wet soil mass (WM). After drying these soil samples in a 60°C drying oven until their weights were stable (24–48 hr), we recorded dry soil mass (DM). We then calculated θ_d_ as (WM – DM)/DM (Figure 2). For each flat, we calculated drying rate as the change in θ_d_ per day between Days 17 and 24. θ_d_ of flats from the well‐watered treatment remained constant or increased over the course of the experiment with a drying rate between 0.010 and 0.305 (mean = 0.164 ± 0.012). Flats from the dry‐down treatment decreased over the course of the experiment, with a drying rate between −0.492 and −0.120 (mean: −0.344 ± 0.013). Measurements were discontinued after Day 32 because the standard deviation of θ_d_ for the dry‐down flats overlapped with zero (Figure 2).

### 2.3 Plant trait measurements

To investigate variation within and between *Mimulus* species for response to water limitation, we quantified a number of drought‐related traits under each watering regime. All temporal values were numbered relative to Day 0, when seeds were transferred into growth chambers following stratification.

#### 2.3.1 Developmental timing

We recorded the dates when plants reached each of three life stages: (a) the production of a bud, (b) the production of an open flower, and (c) the production of a mature fruit. We scored budding date as the first day when the first bud on the primary inflorescence was visible. We scored flowering date as the day when we observed a fully emerged flower displaying a receptive stigma from either the first or second flower pair (*M. guttatus* and *M. nasutus* produce pairs of flowers in sequential progression up flowering stems). Finally, we scored fruiting date as the day when at least one brown, dehiscent fruit containing visible, mobile seeds was produced. The experiment was terminated on Day 63, the day on which the last pollinated flower set seed (see below for pollination details).

#### 2.3.2 Lifetime maternal fitness

We obtained survival and maturation rates by daily inspection of plants. For most *M. guttatus* plants that survived to flowering, we marked and hand‐pollinated one flower, on its first day of stigma receptivity, from the first or second flower pair with pollen donated from the IM767 edge plants (in some cases, we were unable to perform pollinations before plants dropped their corollas; these individuals were dropped from our analyses). IM767, an inbred line derived from the allopatric Iron Mountain population, was used as the common pollen donor as it is likely to be roughly equally differentiated from all CAC samples (pairwise nucleotide diversity, π_s_, is ~ 5% between IM and CAC plants, see Brandvain et al., 2014). Following initial pollinations, the few flowers that remained receptive were hand‐pollinated a second time to ensure pollen was not limiting. For most *M. nasutus* plants that survived to flowering, we marked one or two flowers from the first or second flower pair, and allowed them to self‐fertilize (in some cases, we marked flowers from later pairs; these individuals were dropped from our analyses). From these marked flowers, we measured an individual's seed production on a per fruit basis. We note that hand pollination in *M. guttatus* versus self‐pollination in *M. nasutus* might contribute to species differences in seed production. Nevertheless, variation in seed set due to treatment or species × treatment will be readily detectable.

#### 2.3.3 Rosette diameter

Using calipers, we measured the rosette diameter of plants at their widest points on Day 25.

#### 2.3.4 Floral traits

For most plants that survived to flowering, we measured the corolla length and width of one marked flower on the first or second flower pair (in some cases, plants dropped their corollas before measurements could be taken) on the day it was recorded as flowering. We measured corolla length as the distance from the base of the calyx to the end of the longest petal when hand straightened and corolla width as the distance between the widest point of the bottom petal lobes.

#### 2.3.5 Stomatal density

For plants that survived to Day 52 with healthy green tissue (those in the well‐watered treatment), we made a pressing of the abaxial surface of the largest, fully expanded leaf using GE Clear 100% Silicone Caulk (General Electric). We taped these pressings to slides and examined them under a light microscope. For each leaf, we randomly selected four fields of view at 1000x magnification and counted the number of stomata; we took the average of these four values to compute stomatal density (number of stomata per field of view).

### 2.4 Data analysis

To determine if drying rate of the dry‐down flats varied among locations in the greenhouse, we recorded the position of each flat within an 8 × 13 grid (in north‐to‐south and east‐to‐west directions, respectively; not all positions contained a flat) and performed a multiple regression analysis. The model had “north‐to‐south position” (fixed effect) and east‐to‐west position (fixed effect) as main effects, as well as the two‐way interaction effect. We found that flat position was indeed a significant predictor of drying rate (Multiple regression, *F*_3,320_ = 29.60, *p* < .0001; north–south position: *F* = 33.76, *p* < .0001; east–west position: *F* = 33.68, *p* < .0001; north–south position × east–west position: *F* = 5.96, *p* = .0151). However, we found no significant differences in the drying rate (change per day in θ_d_ between Days 17 and 24) experienced by any particular *Mimulus* line or group under dry‐down conditions (ANOVA with “group” as a fixed effect with “line” nested within it: *F*_18,305_ = 1.0351, *p* = .42). These tests were performed in JMP 13.0 (SAS Institute).

For all drought‐ and fitness‐related traits measured, we used hierarchical ANOVAs to calculate least square means (LSMs) for the three *Mimulus* groups (*M. nasutus*, annual *M. guttatus*, and perennial *M. guttatus*) under each watering treatment. For rosette diameter and seed set, models included “group” (fixed effect with “line” nested within it) and “treatment” (fixed effect) as main effects, “group × treatment” as an interaction effect, and “block” as a random effect. Models were identical for all flower measurements (corolla length, corolla width, days to bud, days bud to flower), but because no perennial *M. guttatus* flowered under dry‐down conditions, only annual *M. nasutus* and annual *M. guttatus* were included in effect tests. The model estimating the number of days from flower to fruit included “group” (fixed effect) and “treatment” (fixed effect) as main effects, “group × treatment” as an interaction effect, and “block” as a random effect (LSMs for annual *M. guttatus* in the dry‐down treatment could not be estimated from models including a nested “line” term due to small sample size of this group). Because it was measured only under well‐watered conditions, the model estimating stomatal density included only “group” (fixed effect, “line” nested within it) and “block” as a random effect. These ANOVAs were run using the lmerTest package in R v. 3.2.3 using a Satterthwaite approximation to account for different variances among groups. We determined significance using a Bonferroni correction of α = .006 (to correct for multiple comparisons) and performed post hoc Tukey–Kramer HSD tests (*p* < .05) on all significant effects.

To investigate variation in seed set within groups, we used JMP 13.0 (SAS Institute) to perform a two‐way ANOVA to calculate LSMs for each plant line; the model included “line” (fixed effect) and “treatment” (fixed effect) as main effects and a “line × treatment” interaction effect. Post hoc *t* tests were used to compare treatments within each line.

To examine the effect of drought across the entire plant life cycle, we calculated the relative decrease in survival for each plant line at each life stage using the following formula: [(proportion individuals surviving under dry‐down) – (proportion surviving under well‐watered)]/ (proportion surviving under well‐watered). Additionally, using JMP 13.0 (SAS Institute), we visualized survivorship to each life stage in CAC *M. nasutus* and *M. guttatus* with Kaplan–Meier Plots and used Cox Proportional Hazards to test for significant differences in maturation rate of each species between treatments. Significant hazards ratios indicate shifts in developmental timing a species exhibited when drought‐stressed as compared to well‐watered individuals.

To investigate the potential for trade‐offs between floral investment and fitness under drought, we conducted linear regression analyses for *M. nasutus* and annual *M. guttatus*. These models tested whether flower size (i.e., average corolla width of each line under well‐watered conditions) affected seed production in the dry‐down treatment. To test for selection on flowering time under drought conditions, we performed a multiple linear regression examining seed set in CAC *M. nasutus* and *M. guttatus* with “days to bud” (fixed effect) and “line” (fixed effect) as main effects, as well as “days to bud × line” as an interaction effect. These analyses were performed using JMP 13.0 (SAS Institute).

## 3 RESULTS

**Figure 3:**
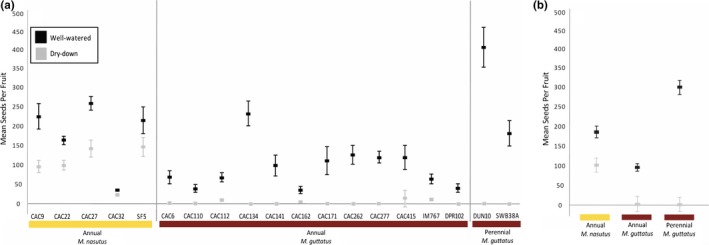
The impact of drought treatment on seed production varies among *Mimulus* line and species/ecotypes. (a) Least squares means, seeds per fruit (*SE*), of experimental lines and (b) least squares means, seeds per fruit (*SE*) of species/ecotypes under each watering regime. Seed production for all lines was significantly lower (*p* < .05, except SF5, *p* < .1, post hoc pairwise *t* tests) and for all species/ecotypes (*p* < .05, post hoc Tukey–Kramer HSD) under simulated drought (gray) than under well‐watered conditions (black), but the reduction was much more severe in *M. guttatus*

Our simulated drought treatment had clear and consistently negative impacts on *Mimulus* growth and fitness, but the effects were not uniform across the three groups (perennial *M. guttatus*, annual *M. guttatus*, and annual *M. nasutus*). Rosette diameter, flower size (corolla width and length), and seed production were all strongly reduced under dry drown conditions (Table 2), but the extent of the reduction in flower size and seed production varied dramatically among groups (i.e., we observed significant “group × treatment” interactions in Table 3). As previously documented (Wu et al., 2010), perennial *M. guttatus* performed particularly poorly: none of the 36 plants exposed to drought‐like conditions survived to produce any flowers (Table 2, Figure 3). Similarly, all annual *M. guttatus* lines (including those derived from the sympatric CAC site), were severely impacted by drought, showing an average reduction in seeds per fruit of 97% under the dry‐down treatment (Table 2, Figure 3). In contrast, *M. nasutus* lines showed only a 42% reduction in seeds per fruit under dry‐down conditions. Taken together, these results demonstrate a dramatic divergence in drought response between *M. guttatus* and *M. nasutus* that persists even in sympatry.

**Table 2:** *Mimulus* species/ecotype least squares means for drought‐ and fitness‐related traits when grown under different watering regimes

Group	Treatment	Rosette Diameter (mm)	Stomatal Density^a^	Corolla Length (mm)	Corolla Width (mm)	Days to Bud	Days Bud to Flower	Days Flower to Fruit	% Survival to Bud	% Survival to Flower	% Survival to Fruit	Seeds per Fruit
perennial *M. guttatus*	WW	63.62 b (2.63, 36)	18.98 a (0.64, 24)	40.39 a (0.55, 36)	33.26 a (0.49, 36)	34.37 a (0.55, 36)	12.74 b (0.41, 36)	14.94 b (0.81, 32)	100	100	92	299.15 a (15.13, 36)
	DD	34.68 d (2.65, 36)	–	–	–	–	–	–	0	0	0	1.36 d (15.18, 36)
annual *M. guttatus*	WW	58.45 bc (1.43, 216)	7.66 b (0.37, 111)	23.74 b (0.30, 180)	18.56 b (0.26, 180)	20.67 b (0.34, 208)	14.05 a (0.22, 196)	23.98 a (0.39, 158)	96	94	77	96.48 c (7.94, 181)
	DD	35.22 d (2.83, 215)	–	14.24 c (0.61, 70)	9.01 c (0.54, 70)	17.49 c (0.61, 179)	9.23 c (0.44, 77)	24.06 a (1.18, 17)	83	37	8	2.78 d (16.22, 72)
*M. nasutus*	WW	88.97 a (2.15, 90)	5.93 c (0.49, 47)	14.84 c (0.45, 59)	7.11 d (0.40, 59)	21.11 b (0.46, 90)	8.96 c (0.33, 90)	25.03 a (0.60, 90)	100	100	100	180.07 b (12.29, 59)
	DD	52.73 c (2.61, 91)	–	11.00 d (0.55, 46)	2.30 e (0.49, 46)	19.52 b (0.56, 82)	8.60 c (0.41, 70)	13.38 b (0.69, 69)	91	79	76	104.17 c (14.98, 46)

Note

Standard error and sample size given in parentheses. Models: Rosette diameter, corolla length, corolla width, days to bud, days bud to flower, and seeds per fruit: “group” (fixed effect with “line” nested within it) and “treatment” (fixed effect), main effects, “group × treatment,” interaction effect “block,” random effect. Days flower to fruit: “group” (fixed effect) and “treatment” (fixed effect), main effects, “group × treatment,” interaction effect, “block,” random effect. Stomatal density: “group” (fixed effect with “line” nested within it) and “block,” random effect. Letters indicate Tukey–Kramer grouping for each trait following ANOVA

a Stomatal Density was only measured on a subset of plants grown under well‐watered conditions.

**Table 3:** Hierarchical ANOVA results for rosette diameter, corolla width, and seed set using a Satterthwaite approximation including “group” (fixed effect with “line” nested within it) and “treatment” (fixed effect) as main effects, “group × treatment” (interaction effect), “block,” random effect. Significance determined using a Bonferroni correction of α = 0.006

	SS	*df*	MS	*F*	*p*
Rosette diameter					
Group	29,481	2	14,740	72.28	<.0001
Treatment	35,633	1	35,633	174.73	<.0001
Group*Treatment	1,889	2	944.41	4.63	.010
Corolla width					
Group	3,600	1	3,600	497.61	<.0001
Treatment	1,804	1	1,804	249.38	<.0001
Group*Treatment	244.93	1	244.93	33.86	<.0001
Seed set					
Group	552,066	2	276,033	39.33	<.0001
Treatment	946,886	1	946,886	134.90	<.0001
Group*Treatment	552,575	2	276,287	39.36	<.0001

Abbreviations: *df,* degrees of freedom; MS, Mean‐Squares; SS, Sum‐of‐Squares.

**Figure 4:**
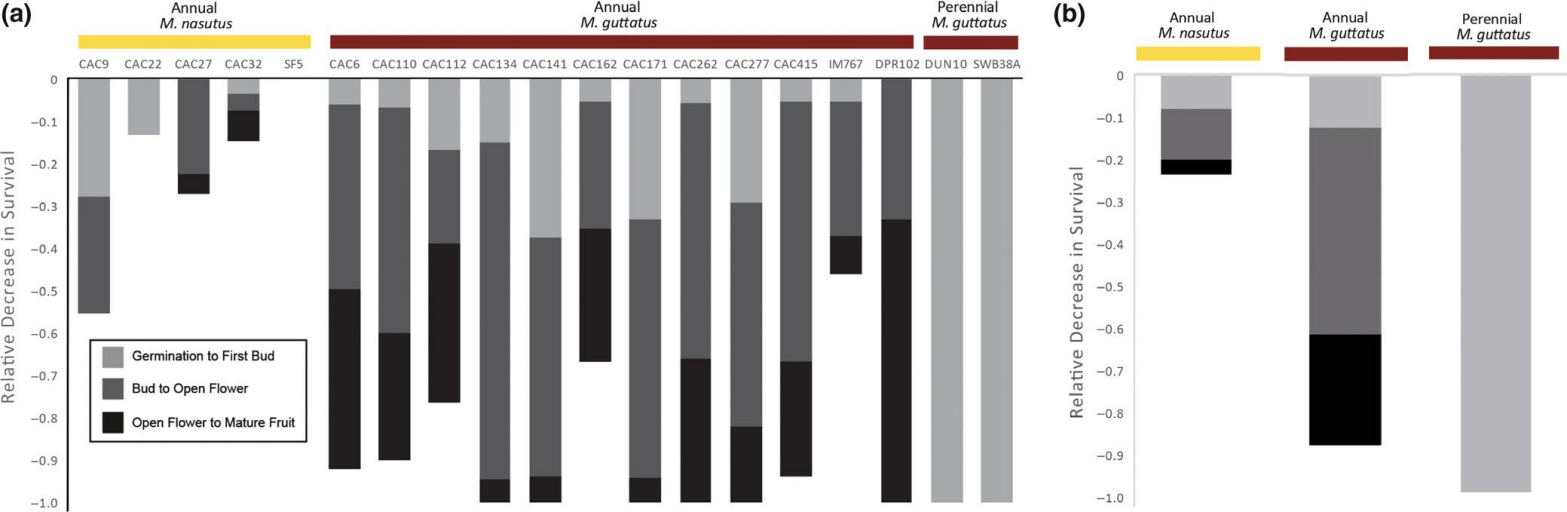
Variation among (a) *Mimulus* lines and (b) species/ecotypes in drought response across the life cycle. Reduction in survival rate in dry‐down versus well‐watered conditions during each of three life stages (germination to first bud, bud to open flower, and open flower to mature fruit)

To explore the phenotypic basis of interspecific differences in seed production under simulated drought, we determined rates of mortality in the two watering regimes during each of three developmental intervals: (a) germination to bud, (b) bud to open flower, and (c) open flower to mature fruit. At each of these life stages, mortality was higher in *M. guttatus* than in *M. nasutus* (Table 2, Figure 4). In perennial *M. guttatus*, which flowers much more slowly than annual *M. guttatus* or *M. nasutus* (i.e., nearly twice as long under well‐watered conditions, Table 2; Twyford & Friedman, 2015; Wu et al., 2010), mortality was complete and occurred early; not a single plant survived long enough to produce a bud. Overall survival rates of annual *M. guttatus* under simulated drought were also low. However, in contrast to perennial lines, annual *M. guttatus* budded relatively quickly under the dry‐down treatment (roughly three days earlier than under well‐watered conditions, Table 2) and most deaths occurred *after* bud initiation (only 9% of plants that produced buds survived to produce fruits, *N* = 179, Table 2). Indeed, for several lines of *M. guttatus* with high rates of mortality under the dry‐down treatment nearly all plants died only after having initiating reproduction (CAC6, CAC110, CAC415, DPR102, Figure 4). In contrast, very few *M. nasutus* plants died after they had produced a mature flower, suggesting this species has diverged for traits that promote fruit maturation even under severe water limitation.

One key question is which phenotypes might explain this difference in late‐stage survival between annual *M. guttatu*s and *M. nasutus*. One possibility is that larger flowers in *M. guttatus* make it more vulnerable to drought. However, we found no evidence for trade‐offs between flower size and fitness under drought in either species; lines with larger flowers (i.e., wider corollas) under well‐watered conditions showed no deficit in seed set under dry‐down conditions (Linear regression, *M. guttatus: R* ^2^ = .03, slope = 0.35, *M. nasutus: R* ^2^ = .06, slope = −7.18). It is also possible that key vegetative traits might differ between the two species. Indeed, we found that annual *M. nasutus* had significantly lower stomatal density (a trait often associated with higher water use efficiency) than leaves from *M. guttatus* (Table 2).

**Figure 5:**
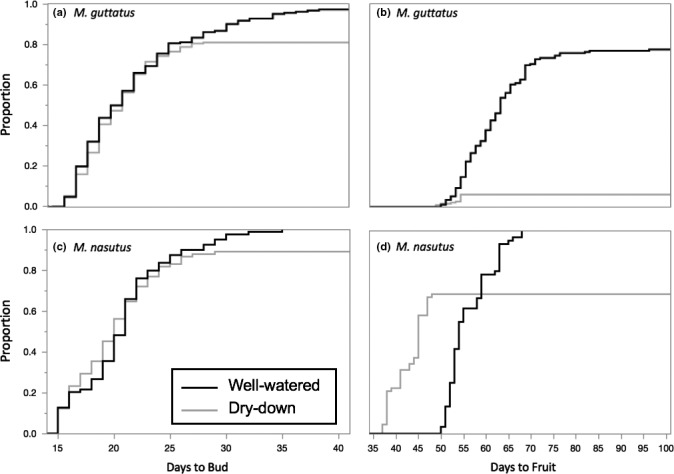
Divergence in response to experimental drought between sympatric *M. guttatus* and *M. nasutus* was more pronounced later in the life cycle. Kaplan–Meier Plots showing progression from germination to bud (a, c) and to mature fruit (b, d) of Catherine Creek *M. guttatus* and *M. nasutus* under well‐watered (black) and dry‐down (gray) conditions. Days are numbered relative to Day 0, when seeds were transferred into growth chambers following stratification

**Figure 6:**
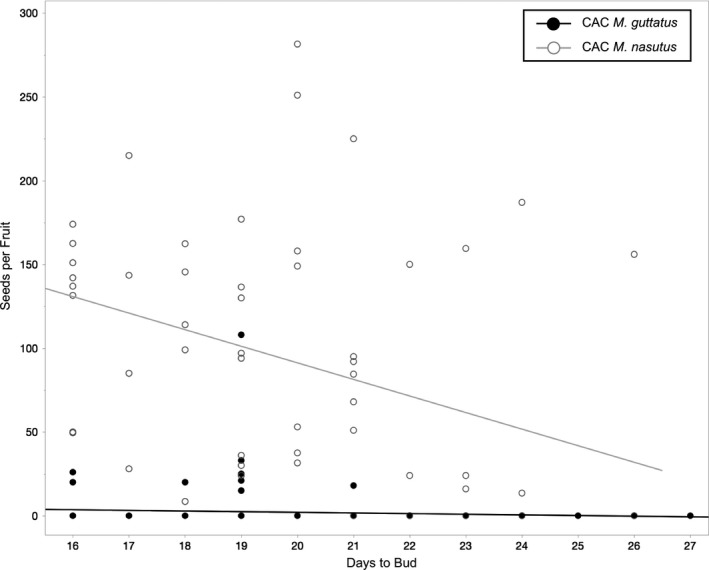
Under simulated drought, the effect of flowering time on seed production varies between sympatric *Mimulus* species. In a multiple linear regression (quadratic and cubic regression showed lower support), days to bud and plant line were significant predictors of seeds production in *M. nasutus* (“days to bud”: *F* = 6.14, *p* = .0166; “line”: *F* = 7.18, *p* = .0004; “days to bud × line”: *F* = 0.61, *p* = .6145; *F*_7,49_ = 4.95, *p* = .0003, *R* ^2^ = .41), but not in *M. guttatus* (*F*_19,129_ = 0.64, *p* = .8708, *R* ^2^ = .09)

Restricting our focus to just the sympatric taxa at CAC, it is clear that interspecific differences in drought response become more pronounced later in the life cycle. Under the long days of our experiment, the two species’ flowering phenologies were almost entirely overlapping, regardless of treatment (Figure 5). The one exception to this pattern is that CAC *M. guttatus* budded slightly earlier (i.e., less than a day on average) under dry‐down than under well‐watered conditions (hazards ratio = 0.19, *p* = .0011; Figure 5). However, this very small head start in *M. guttatus* seems to have made little difference in terms of fitness: even the earliest flowering CAC *M. guttatus* usually died before making mature fruits or producing seeds (Figures 4 and 6). In CAC *M. nasutus*, on the other hand, dry‐down seed production was negatively correlated with flowering time (*F* = 6.14, *p* = .0166, Figure 6), showing that this species experiences selection for early flowering in water‐limited environments. In contrast to flowering time, we observed striking differences between CAC *M. guttatus* and *M. nasutus* in fruit maturation rates under dry‐down conditions (Figure 4). Remarkably, *M. nasutus* fruits matured more than 12 days earlier under simulated drought than under well‐watered conditions (hazards ratio = 12.50, *p* < .0001, Figure 5). The late‐stage drought response in CAC *M. guttatus* was very different: among the few plants that survived to produce fruit, maturation occurred only one day earlier than among their well‐watered counterparts (hazards ratio = 1.00, *p* < .0001, Figure 5). Taken together, these results suggest the large differences between CAC *M. guttatus* and *M. nasutus* in dry‐down survival (Figure 4) and seed set (Figure 3) are driven by divergence in postflowering developmental rate.

## 4 DISCUSSION

Previous work has shown that divergence in seasonal flowering behavior is a major component of premating isolation between sympatric *M. nasutus* and *M. guttatus* (Kenney & Sweigart, 2016; Martin & Willis, 2007). At CAC, this phenological shift—with *M. nasutus* flowering earlier in the season than *M. guttatus—*is caused, in part, by species divergence in photoperiod response (Fishman et al., 2014), a change that has undoubtedly facilitated drought escape in *M. nasutus* and allowed it to occupy drier microsites. In this study, we explore phenotypes beyond critical photoperiod that might contribute to microhabitat divergence between sympatric *Mimulus* species. Under inductive conditions, typical of mid‐ to late‐season day lengths, when there is substantial phenological overlap between species at CAC (Kenney & Sweigart, 2016), we find dramatic differences between *M. nasutus* and *M. guttatus* in drought response. Consistent with the natural microhabitats they occupy, CAC *M. nasutus* had much higher fitness than CAC *M. guttatus* under water‐limited conditions, indicating divergence in drought response traits other than the critical photoperiod requirement. Surprisingly, this fitness difference was not due to flowering time; under 16‐hr days, the two species initiated reproduction at roughly the same time in both well‐watered and dry‐down conditions. Instead, differential fitness under drought was largely caused by differences in mortality *after* the onset of flowering, with *M. guttatus* dying at a much higher rate. Higher survival of *M. nasutus* was mediated, at least in part, by a plastic increase in the speed of late‐stage development, particularly during fruit maturation. Discovering the mechanistic basis of this plastic drought response will require additional investigation, but it is likely a key component of species divergence in microhabitat adaptation at CAC.

Although an increase in developmental rate is a hallmark of the drought escape strategy (Ludlow, 1989), few studies investigate time points after bud/flower initiation. Here, if we had restricted our measurements to flowering time, we would not have detected any difference between *Mimulus* species in drought‐induced developmental rate, which arose only after anthesis. Under our long‐day experimental conditions, which effectively removed critical photoperiod as a signal for flowering, CAC *M. guttatus* and *M. nasutus* initiated reproduction at similar rates. Additionally, we found little evidence for plasticity in flowering time as an adaptive response to drought. In fact, only *M. guttatus—*the less drought‐adapted species—mounted a weak plastic response, flowering slightly earlier (less than a day on average) under dry‐down conditions. These results are largely consistent with previous greenhouse studies of drought response using similar inductive conditions (Ivey & Carr, 2012; Wu et al., 2010). Both studies found only modest differences in the intrinsic rate of flowering between *M. nasutus* and *M. guttatus* and little evidence for plastic shifts in flowering time under drought (but see Ivey & Carr, 2012, which found a slight decrease in flowering time for *M. nasutus* under drought conditions). In contrast to these greenhouse studies, plasticity in flowering time has been observed in field transplant experiments involving *M. guttatus* and a closely related selfing species, *M. lacinatus*, which specializes on dry, granite outcrops (Ferris & Willis, 2018).

An important question is whether differences among studies in flowering time and plasticity are due to genetic/phenotypic variability among *Mimulus* populations/species or due to experimental differences. Compared to Wu et al. (2010), plants in our study flowered more rapidly (mean days to flower in perennial *M. guttatus*, annual *M. guttatus*, and annual *M. nasutus* is shifted earlier by ~10 days), potentially due to modest levels of light contamination (see Methods) and/or additional environmental variables (e.g., greenhouse temperature, light intensity). Because of this earlier flowering, we also began our dry‐down treatment nine days sooner than in Wu et al. (2010). Thus, timing of the treatments in the two studies was similar relative to flowering (i.e., dry‐down treatments started ~10 days before the average date of first flowering in *M. nasutus*), suggesting plants experienced drought at similar developmental stages. Of course, in any of these studies, differences in the timing or intensity of drought relative to plant development, or in other environmental variables (e.g., temperature), might affect a plant's ability to mount a plastic response.

Despite the negligible contribution of flowering time to CAC *Mimulus* species differences in drought response, we did find evidence that water limitation imposes selection for early flowering in *M. nasutus*. This result mirrors what has been seen in annual *M. guttatus* subjected to drought in greenhouse experiments (Ivey & Carr, 2012; Wu et al., 2010) and under natural conditions in the field (Ferris & Willis, 2018; Hall & Willis, 2006; Mojica et al., 2012). Indeed, in the alpine Iron Mountain population of annual *M. guttatus*, selection for rapid flowering to escape summer drought trades off with selection for larger flowers, which produce more seeds, but make plants more vulnerable to desiccation (Mojica et al., 2012; Troth, Puzey, Kim, Willis, & Kelly, 2018). In our experiment, because of extremely high mortality in water‐limited *M. guttatus*, we had little power to detect selection for early flowering (very few individuals survived to produce seeds). This level of drought‐induced mortality was much higher than what has been observed for annual *M. guttatus* in previous studies (Ivey & Carr, 2012; Wu et al., 2010), which might be due to CAC‐specific traits. Alternatively, the difference might be explained by variation among experimental conditions: our dry‐down treatment was applied earlier than that of Wu et al., 2010 and was likely more severe than the simulated drought used by Ivey & Carr, 2012.

Given our finding that CAC *Mimulus* species differ dramatically in postflowering mortality under drought, a key question is which specific phenotypes are involved in this divergent response. At least part of the answer is that *M. nasutus* alone responded plastically to the dry‐down conditions, increasing its rate of fruit maturation and setting seed prior to senescence. However, it is not yet clear whether this late‐stage drought response was due to particular traits expressed only later in the life cycle or to some threshold requirement for severe water limitation (which, in our experiment, just happened to coincide with late stages of development). Although traits that promote rapid development to escape drought often show trade‐offs with traits for avoidance (e.g., WUE; Geber & Dawson, 1990; Kenney, McKay, Richards, & Juenger, 2014; McKay, Richards, & Mitchell‐Olds, 2003), previous work in *M. nasutus* and annual *M. guttatus* suggests that the two strategies are not mutually exclusive (Ivey & Carr, 2012; Kooyers et al., 2015; Wu et al., 2010). Thus, it is possible that drought adaptation in *M. nasutus* involves both faster development and traits for avoidance like lower stomatal density that may lead to decreased water loss under drought conditions (Franks, Kane, O'Hara, Tittes, & Rest, 2016; Masle, Gilmore, & Farquhar, 2005). Furthermore, while CAC *M. guttatus* generally wilted under drought conditions, *M. nasutus* remained erect and turgid, and seemed to hasten senescence. The ability to undergo osmotic adjustment to maintain turgor is normally associated with drought tolerance (Chaves, Maroco, & Pereira, 2003), but plants might also be able to avoid the negative consequences of drought by accumulating stores of nutrients when water is plentiful and/or reallocating carbohydrate resources during initial water deficits (Kooyers, 2015). This adaptive response to drought has been well documented in cereal crops (Palta, Kobata, Turner, & Fillery, 1994; Schnyder, 1993; Yang, Zhang, Huang, Zhu, & Wang, 2000) and has also been observed in the Mediterranean annual *Lupinus albus*, which diverts resources from stems to seed pods as soon as it senses drought (Rodrigues, Pacheco, & Chaves, 1995). Going forward, if we are to achieve a more mechanistic understanding of divergence in drought response between CAC *M. nasutus* and *M. gutattus*, future experiments should investigate a more comprehensive set of physiological, leaf, and whole‐plant traits.

In addition to elucidating the mechanisms of drought response within *Mimulus* species, our study provides important insight into the role of differential habitat adaptation in species divergence. Our results suggest that a simple shift in critical photoperiod (from long‐ to short‐day flowering) would be insufficient for CAC *Mimulus* to succeed in microhabitats that dry out sooner in the season. Soil moisture is highly heterogeneous at CAC and although short‐day flowering might enable some plants to complete reproduction before they experience any water limitation, other individuals are likely to occupy patches that impose significant drought stress. As we have seen, CAC *M. nasutus* alone is able to cope with such conditions, surviving longer and speeding up its development to produce many more seeds than CAC *M. guttatus*. The picture emerging from this and previous studies (Ivey & Carr, 2012; Wu et al., 2010) is that habitat divergence between *M. nasutus* and *M. guttatus* is complex, involving many traits, both constitutive and plastic. Although some of the key traits involved might be genetically simple (e.g., critical photoperiod: Fishman et al., 2014), the microhabitat isolation we observe at CAC is likely to involve changes at many loci.

These findings have important implications for species maintenance in sympatry. At CAC and other sympatric sites, introgression is ongoing and asymmetric, with most interspecific gene flow occurring from *M. nasutus* into *M. guttatus* (Brandvain et al., 2014; Kenney & Sweigart, 2016; Sweigart & Willis, 2003). Thus, it is conceivable that in drier years or microsites, introgression of drought response alleles from *M. nasutus* (e.g., for faster fruit maturation) might prove adaptive in *M. guttatus*, allowing it to survive in environments beyond its normal limits. In hybridizing sunflowers, for example, adaptive introgression of drought escape traits from *Helianthus debilis* seem to have facilitated range expansion of *H. annuus* into drier areas (Whitney, Randell, & Rieseberg, 2010). On the other hand, our results suggest that introgression of early‐flowering *M. nasutus* alleles at the two major photoperiod loci (Fishman et al., 2014) might not allow *M. guttatus* to invade drier microsites at CAC; even when *M. guttatus* flowers early, it is unable to overcome water deficits to set seed. This result might help explain why one of the two photoperiod loci remains highly divergent between species (Kenney & Sweigart, 2016), even in the face of considerable interspecific gene flow. Consistent with the idea that differentially adapted loci contribute to reproductive isolation between species, we find evidence of selection against *M. nasutus* ancestry across the *M. guttatus* genome at CAC (Brandvain et al., 2014; Kenney & Sweigart, 2016). Our work here sets the stage for future experiments to map the genetic basis of key ecological traits and fitness across the complex and variable environments of CAC, an approach that holds great promise for understanding how the process of abiotic adaptation can contribute to speciation.

## CONFLICT OF INTEREST

The authors declare no conflicts of interest.

## AUTHOR CONTRIBUTIONS

Research conceived and designed by ALS, data collected and analyzed by SJM, manuscript written by SJM and ALS.

## Data Availability

Data available from the Dryad Digital Repository: https://doi.org/10.5061/dryad.6dh476h
